# Heparins attenuated histone-mediated cytotoxicity in vitro and improved the survival in a rat model of histone-induced organ dysfunction

**DOI:** 10.1186/s40635-015-0072-z

**Published:** 2015-12-29

**Authors:** Toshiaki Iba, Naoyuki Hashiguchi, Isao Nagaoka, Yoko Tabe, Katsuhiko Kadota, Koichi Sato

**Affiliations:** Department of Emergency and Disaster Medicine, Graduate School of Medicine, Juntendo University, 2-1-1 Hongo Bunkyo-ku, Tokyo, 113-8421 Japan; Department of Host Defense and Biochemical Research, Graduate School of Medicine, Juntendo University, 2-1-1 Hongo Bunkyo-ku, Tokyo, 113-8421 Japan; Department of Clinical Laboratory Medicine, Graduate School of Medicine, Juntendo University, 2-1-1 Hongo Bunkyo-ku, Tokyo, 113-8421 Japan; Department of Surgery, Juntendo Shizuoka Hospital, Graduate School of Medicine, Juntendo University, 129 Izunokuni, Shizuoka, 410-2295 Japan

**Keywords:** Histone, Unfractionated heparin, Low-molecular weight heparin, Argatroban

## Abstract

**Background:**

The beneficial effects of heparin in the treatment of severe sepsis, septic shock, and sepsis-associated disseminated intravascular coagulation (DIC) have recently been reported. However, the mechanisms underlying the therapeutic benefits of heparin in these conditions have not yet been clearly elucidated. The purpose of this study was to confirm the effect of heparin of neutralizing histone toxicity.

**Methods:**

Rat models of histone H3-induced organ dysfunction were administered in a low or high dose of unfractionated heparin (UFH), low-molecular-weight heparin (LMWH), or argatroban, and the therapeutic effects of each anticoagulant were examined. In another series, the survival of the histone H3-administered animals was evaluated. Furthermore, the effect of each of the aforementioned anticoagulants on cell death induced by histone H3 was examined in cultured vascular endothelial cells and leukocytes.

**Results:**

Although UFH, LMWH, and argatroban significantly suppressed the histone-induced decrease of the WBC and platelet counts in the animal models of organ dysfunction, only UFH and LMWH attenuated hepatic and renal dysfunction. In addition, the mortality was significantly reduced only by high-dose UFH and LMWH. The in vitro study revealed that both vascular endothelial cell death and leukocyte cell death were significantly attenuated by UFH and LMWH but not by argatroban.

**Conclusions:**

The histone-neutralizing effect of heparin may contribute to the beneficial effects of heparins observed in the animal study. The results of the in vitro study further confirmed the above contention and suggested that heparin binds to histones to attenuate the cytotoxic actions of the latter*.* Since heparin has been demonstrated to protect animals from the organ damage induced by histones and consequently reduce the mortality, administration of heparin could become a treatment of choice for patients suffering from severe sepsis.

## Background

Extracellular histones released from dead cells play important roles in cellular damage in sepsis [1]. Xu et al. [2] reported that the presence of extracellular histones was associated with endothelial cytotoxicity, organ failure, and death in animal models of sepsis. Ekaney et al. [3] have demonstrated the increased levels of circulating histone in septic patients. They also reported that histones play important roles in the pathogenesis of sepsis. Based on the above knowledge, histones are considered as attractive therapeutic targets for future studies.

There has been a long-standing debate on the possible usefulness of heparin in the management of patients with severe sepsis [4, 5]. As the existence of close connections was revealed between activation of the coagulation cascade and the development of organ dysfunction in sepsis [6, 7], strategies aimed at inhibition of coagulation were developed and found favor in experimental and clinical studies [8]. Subsequently, a randomized controlled trial (RCT) was carried out to examine the effect of low-dose unfractionated heparin (UFH), which inhibits the coagulation system without increasing the bleeding risk, as a complementary treatment for sepsis [5, 9]. Although these studies failed to yield the expected results, recent systematic reviews have reported a consistent trend of favorable results. Zartcgabski et al. [10] reported that the risk hazard ratio for death associated with the use of heparin was 0.88 (95 % confidence interval (CI), 0.77–1.00; *I*^*2*^ = 0 %). In addition, Wang et al. [11] also reported a decreased mortality associated with heparin use (odds ratio =0.656, 95 % CI =0.562–0.765, *p* < 0.0001). Although the trend was consistent, it would be important to bear in mind the differences in the types of heparin preparations used in these clinical studies, as different heparin preparations may exert different effects. In Wang’s analysis, 40 % of the cases were treated with UFH and the rest with either UFH or low-molecular-weight heparin (LMWH) [11]. In Zartcgabski’s analysis, 11 % of the cases were treated with UFH, while the rest were treated with LMWH, both UFH and LMWH, or a combination of either UFH or LMWH with activated protein C [10]. However, few studies have been conducted to examine the differences in the effects of different heparin formulations, especially in relation to the effect on neutralizing histone toxicity. In this study, we attempted to compare the effect of UFH, LMWH, and argatroban [12]. In the first experiment, the effect of each anticoagulant was examined in animal models of histone-induced organ dysfunction, and in the second experiment, the effects of the anticoagulants were examined in a coagulation factor-free in vitro setting.

Argatroban is a synthetic direct thrombin inhibitor and is often used as an anticoagulant [13]. Based on the aforementioned theory, argatroban may be expected to be useful in the treatment of sepsis based on its effect of inhibiting thrombin. Fuchs et al. [14] examined the effect of argatroban on peritonitis-induced impairment of microcirculation in a rat model of sepsis and reported improvement of the intestinal microcirculation; they attributed this effect to reduced leukocyte adherence to the endothelium induced by the drug. Thus, another purpose of this study was to also examine the effect of argatroban in the same model.

## Methods

### Animal experiment

Ten-week-old male Wistar rats were used for this study. All experimental procedures were performed after obtaining the approval of the Ethical Committee for Animal Experiments of Juntendo University. All the experimental rats were provided with standard rat chow and water ad libitum. In the first experiment, the rats were anesthetized with sodium pentobarbital (40 mg/kg, intraperitoneally), and 36 animals were divided into three groups, as follows: the UFH group, assigned to receive 350 (low dose) or 700 (high dose) U/kg of UFH (heparin sodium, Mitsubishi-Tanabe Pharma Co., Osaka, Japan) by intravenous injection (*n* = 6 in each subgroup); the LMWH group, assigned to receive 2.0 (low dose) or 4.0 (high dose) mg/kg of enoxaparin (Sanofi Aventis, Paris, France) by intravenous injection (*n* = 6 in each subgroup); and the argatroban group, assigned to receive 3.6 (low dose) or 7.2 (high dose) mg/kg of argatroban by intravenous injection (*n* = 6 in each subgroup). The doses of each of the anticoagulants were set so as to obtain comparable anticoagulant effects [15]. Immediately after the administration of each anticoagulant, the animals were administered 50.0 mg/kg of histone H3 (Calf thymus histone H3, Sigma-Aldrich, Co. (St. Louis, MO, USA)) by intravenous injection. An additional group of animals (*n* = 6), which served as the control group, was given saline and histone H3.

Blood samples were obtained from the inferior vena cava 6 h after the administration of histone H3. The white blood cell (WBC) and platelet counts were determined using an automated device for animals (Celltac, MEK-5128; Nihon Kohden Co., Ltd., Tokyo, Japan). Citrated plasma samples were obtained by whole blood centrifugation and utilized for the assays; the levels of fibrin/fibrinogen degradation products (FDP), alanine aminotransferase (ALT), and blood urea nitrogen (BUN) were measured in these samples. The FDP levels were determined using an enzyme-linked immunosorbent assay kit (Teikoku Laboratories, Tokyo, Japan).

In another series, the survival of the rat models of histone H3 (25, 50, 100 mg/kg)-induced organ dysfunction was examined. At 24 h after the administration of histone H3, the survival was calculated in the animal subgroups that had been treated with the higher dose of each agent (*n* = 6, in each subgroup).

### In vitro experiment

Rat aortic endothelial cells were purchased from Cell Applications, Inc. (San Diego, CA, USA). Endothelial cells were routinely cultured in Dulbecco’s modified Eagle’s medium (DMEM, Invitrogen **#**22320022 containing 2 mM l-glutamine, 0.1 mM non-essential amino acids, 100 U/mL of penicillin, and 100 mg/mL of streptomycin) supplemented with 10 % fetal bovine serum (FBS, Gibco, NY, USA). For the experiments, the endothelial cells were seeded in six-well tissue culture plates at 10 × 10^6^ cells/well in DMEM supplemented with 10 % FBS and grown to confluence. Cells were washed in two changes of phosphate-buffered saline (PBS, Gibco) before being used for the experiments, and the culture medium was changed to Opti-MEM (Life Technologies, Carisbad, CA, USA) not containing FBS. Histone H3 was added to the medium to a final concentration of 25 or 50 μg/mL.

To evaluate cell death, the endothelial cells were observed using the Eclipse Pol microscopic system (Nikon Co., Ltd., Tokyo, Japan) at 6 h after the histone H3 administration. To calculate the cell viability, the culture wells were immersed in 50 % ethanol at 37 °C for 1 h and 100 % methanol at 37 °C for 1 h, and then stained with 1 μg/ml of 4′,6-diamidino-2-phenylindole (DAPI, 0.01 mg/ml in Tris-EDTA buffer solution containing 10 mM 2-mercaptoethylamine (pH 7.4), to visualize the DNA. Endothelial cells were also stained with Apoptosis/Necrosis Detection Kit (ENZ-51002-25, Enzo Life Sciences, NY, USA) according to the manufacturer’s instructions. Three fields in each well were examined by fluorescence microscopy by two individuals, and the mean ratio of the count of unstained cells to that of the total cell count was calculated as the cell viability.

Leukocytes obtained from healthy rats were utilized for the examination of cell death. Rats were anesthetized and given an intraperitoneal injection of 10 ml of 1 % oyster glycogen Type II (Sigma-Aldrich Co., St. Louis, USA) in PBS. Six hours later, the peritoneal cavities were washed with 10 mL of RPMI 1640 (Gibco, Carlsbad, USA). The sampled peritoneal lavage fluid was centrifuged at 400×*g* for 5 min and then washed with the collection buffer. Then, the leukocytes were resuspended in 500 μL of the culture medium. Following the same steps as those adopted for the endothelial cells, the leukocyte survival at 6 h after the histone H3 administration was calculated.

### Statistical analysis

All the data are expressed as mean ± standard deviation. Statistical analysis was performed using one-way analysis of variance for comparison of the means, using the StatView II statistical software package for Macintosh. To compare the differences in the survival between the groups, the chi-square test was used. Statistical differences were deemed significant at a level of *p* < 0.05.

## Results

### Animal experiment

**Fig. 1:**
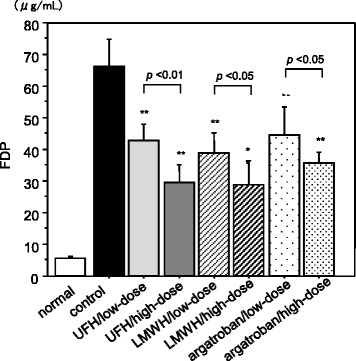
In vivo effects of unfractionated heparin, low-molecular-weight heparin, and argatroban. Significant decreases of the fibrin/fibrinogen degradation products (FDP) were observed in all of the treatment groups as compared to the level in the histone H3-treated control. Similar effects were observed in the high-dose and low-dose groups of each agent. The animals given with histone H3 and saline were served as control. Blood samples were obtained 6 h after the administration of histone H3. Unfractionated heparin (UFH), low dose 350 U/kg, high dose 700 U/kg; low-molecular-weight heparin (LMWH), low dose 2.0 mg/kg, high dose 4.0 mg/kg; argatroban, low dose 3.6 mg/kg, high dose 7.2 mg/kg. **p < 0.01 (*n* = 6 in each subgroup)

Significant reductions of the elevated FDP level as compared to that in the control group was observed in all of the high- and low-dose UFH, LMWH, and argatroban groups (*p* < 0.01, respectively). The FDP levels were significantly lower in the high-dose groups than in the low-dose groups (*p* < 0.01 for the UFH group, and *p* < 0.05 for the LMWH and argatroban groups) (Fig. 1).

**Fig. 2:**
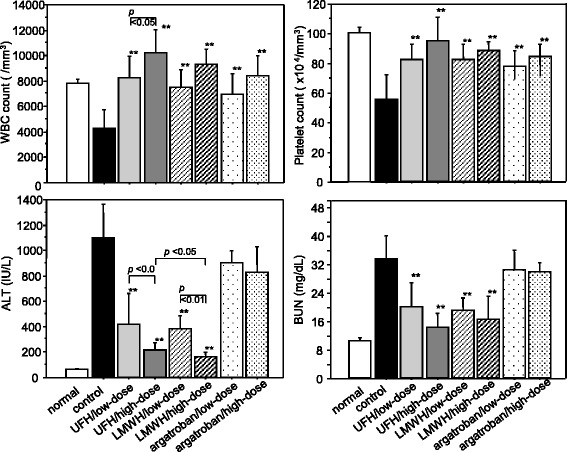
In vivo effects of unfractionated heparin, low-molecular-weight heparin, and argatroban. Decreases of the white blood cell (WBC) and platelet counts by histone H3 were significantly suppressed by all of the anticoagulants. Increased alanine aminotransferase (ALT) and blood urea nitrogen (BUN) levels were significantly suppressed by unfractionated heparin (UFH) and low-molecular-weight heparin (LMWH); however, no such effect was observed with argatroban. The animals given with histone H3 and saline were served as control. Blood samples were obtained 6 h after the administration of histone H3. UFH, low dose 350 U/kg, high dose 700 U/kg; LMWH, low dose 2.0 mg/kg, high dose 4.0 mg/kg; argatroban, low dose 3.6 mg/kg, high dose 7.2 mg/kg. **p < 0.01 (*n* = 6 in each subgroup)

Evaluation of the cell counts demonstrated that the WBC counts were significantly better maintained in all of the UFH, LMWH, and argatroban groups, with the effect being more prominent in the high-dose UFH subgroup than in the low-dose UFH subgroup (Fig. 2, upper left). Similarly, the platelet counts were also significantly better maintained in all of the treatment groups (Fig. 2, upper right), although no difference in the platelet count between the high-dose and low-dose subgroups was recognized for any anticoagulant. Organ damage markers represented by ALT and BUN were significantly lower in the UFH and LMWH groups as compared to the control group. The ALT level was 1097 ± 272 IU/L in the control group, 207 ± 63 IU/L in the high-dose UFH subgroup, and 168 ± 32 IU/L in the low-dose UFH subgroup. The ALT level in the high-dose LMWH subgroup was lower than that in the high-dose UFH subgroup (*p* < 0.05). In contrast to the findings in the UFH and LMWH groups, no significant difference in the ALT level was found in the argatroban group (Fig. 2, lower left). Elevation of the BUN was similarly attenuated in the UFH and LMWH groups, while no such effect was recognized in the argatroban group (Fig. 2, lower right).

With respect to the survival, one out of six animals (17 %) administered with 25 mg/kg of histone H3 survived, while none (0 %) of the animals with administered 50 or 100 mg/kg of histone H3 survived. On the other hand, following treatment with high-dose UFH, all of the six animals administered with 50 mg/kg histone H3 survived (*p* < 0.01). High-dose LMWH administration also improved the survival (6/6, 100 %, *p* < 0.05) in the animals given 50 mg/kg of histone H3 (Table 1).

**Table 1:** Comparison of survival rates in a rat model of histone H3 administration

Group	Histone H3 dose (mg/kg)		
	25	50	100
No treatment	1/6 (17 %)	0/6 (0 %)	0/6 (0 %)
UFH high-dose	6/6* (100 %)	6/6** (100 %)	4/6 (67 %)
LMWH high-dose	6/6* (100 %)	5/6* (83 %)	4/6 (67 %)
Argatroban high-dose	1/6 (17 %)	1/6 (17 %)	0/6 (0 %)

*p < 0.05; **p < 0.01 compared with no treatment group

### In vitro experiment

**Fig. 3:**
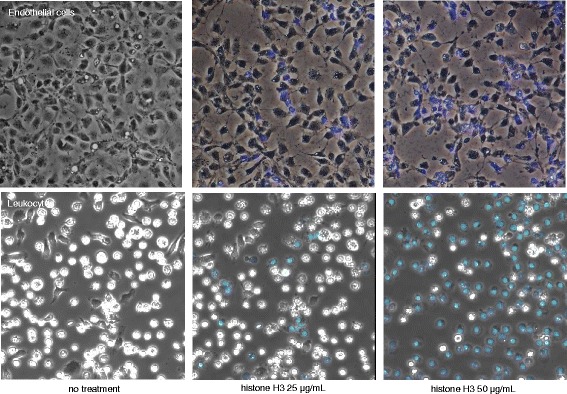
In vitro effects of histone H3 on endothelial cell and leukocytes. The figure depicts the merged phase-contrast and immunofluorescence images. Endothelial cell and leukocyte viability were observed at 6 h after the histone H3 administration. The rat aortic endothelial cells (*upper panels*) and leukocytes harvested from the abdominal cavity (*lower panels*) were incubated with calf thymus histone (25 [*middle*] and 50 [*right*] μg/mL). The nuclei of the dead cells were stained *blue* with DAPI (4′,6-diamidino-2-phenylindole) (objective lens ×20)

Figure 3 upper panels show the merged phase-contrast and fluorescence images of endothelial cells at the indicated histone H3 doses. Histone H3 at the concentration of 25 μg/mL caused remarkable shrinkage and separation of the cell-cell junctions at 6 h. Endothelial cell death was confirmed by the uptake of DAPI that binds to DNA in the nucleus through the cellular membranes that show one small diffuse fluorescent punctum per cell. Small numbers of cells were stained following treatment with 25 μg/mL of histone H3, while the blue puncta clearly increased in the cells treated with 50 μg/mL of histone H3. Figure 3 lower panels show the merged phase-contrast and fluorescence images of selected leukocytes (more than 90 % of the leukocytes are neutrophils) at the indicated histone H3 doses. Histone H3 at the concentration of 50 μg/mL caused remarkable leukocyte cell-death confirmed by the uptake of DAPI at 6 h.

**Fig. 4:**
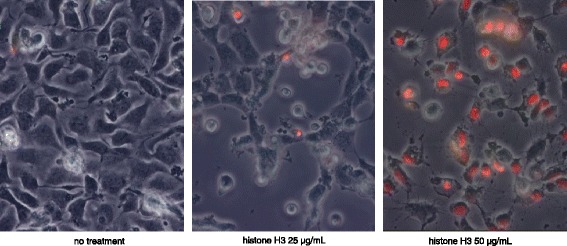
In vitro effects of histone H3 on endothelial cell. Merged phase-contrast and immunofluorescence images of the rat aortic vascular endothelial cells. Confluent monolayers of cells (*left*) were incubated with calf thymus histone (25 [*middle*] and 50 [*right*] μg/mL). The endothelial cells shrank and became sparse at 6 h after treatment with 50 μg/mL of histone H3. The endothelial cells death was detected microscopically by 7-amino actinomycin D (7-AAD, *red*) (objective lens ×40)

Figure 4 depicts the morphological change and cell-death of the endothelial cell. Many of the cells treated with 50 μg/mL of histone H3 were stained with 7-amino actinomycin D (7-ADD, red).

**Fig. 5:**
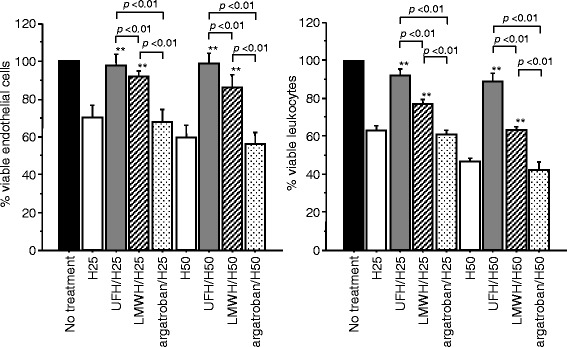
In vitro effects of unfractionated heparin, low-molecular-weight heparin, and argatroban. The survival of the endothelial cells at 6 h after histone H3 treatment was maintained better with UFH and LMWH but not with argatroban (*left*). Similarly, the survival of leukocytes was maintained better only with UFH and LMWH (*right*)

The survival of the endothelial cell was significantly maintained better under the treatment with UFH and LMWH. However, such effect was not recognized with argatroban. (Fig. 5, left). Similarly, the leukocyte survival was better maintained under the treatment with UFH and LMWH. No such effect was recognized with argatroban (Fig. 5, right).

## Discussion

Neutrophils are the first-line defenders against microorganisms. They protect the host against invading pathogens by phagocytosis and by releasing antimicrobial peptides and proteolytic enzymes, as well as by inducing the production of reactive oxygen species [16]. In addition to these mechanisms, the newly discovered host defense mechanism, neutrophil extracellular traps (NETs), has attracted enormous attention [17]. NETs are composed of DNA in association with granular proteins, such as elastase and cathepsin G, and some other cytoplasmic proteins and histones. Saffarzadeh et al. [18] reported that histones play the most important roles among them. Although histones attached to the decondensed nuclear chromatin are considered to be responsible for the microorganism killing, circulating histones detached from chromatin act as danger-associated molecular patterns (DAMPs) [19]. In this context, histones are recognized as particularly harmful inflammatory mediators [3, 20, 21]. Xu et al. [2] reported that histones H3 and H4 are the major cytotoxic effectors among the histones, whereas Bosmann et al. demonstrated histone H4 in bronchoalveolar lavage fluid (BALF) played the key role in the development of acute lung injury [22]. We still do not know enough about which histone plays a major role in the pathophysiology of severe sepsis. Though we pick up histone H3 in the present experiment, the roles of other histones, especially the role of histone H4, should be examined in the future study. As for the mechanism of histone neutralization, it is hypothesized that since histones are positively charged nuclear proteins, their cytotoxicity could be reduced by polysialic acids, such as heparin [18]. Heparin, consisting of a high volume of negatively charged sulfated proteoglycans, binds to histones and inactivates them through high-affinity electrostatic interactions [23]. One of the purposes of this study was to examine the organ- and life-protective effects of UFH and LMWH, and our results indicated that both heparins demonstrated remarkable effects in both the in vitro and in vivo studies. However, ours is not the first report on the protective effect of heparin. Wang et al. [24] reported that coinjection of a low dose of heparin with a lethal dose of histones protected mice from organ damage and death. Another purpose of this study was to compare the effects of UFH and LMWH. Initially, we speculated that the effects of UFH would be more prominent as compared to those of LMWH, because of the structural and metabolic pathway differences. UFH contains larger numbers of sulfates and has a shorter half-life. Thus, for comparable anticoagulant activity, the net negative charge of UFH might be larger than that of LMWH. However, no such superiority of UFH was recognized in the animal study, and the liver function was actually maintained better in the LMWH group. In contrast, the protective effect against cell death was more evident in the UFH group. We cannot explain this discrepancy clearly; however, we assume that this result may be related to the differences in the metabolic pathway between the two heparin preparations.

With regard to the anticoagulant effect dependency, Wildhagen et al. [25] demonstrated the fractioned non-anticoagulant heparin suppresses histone cytotoxicity and reduces the mortality of mouse models of sepsis. We expected that the anticoagulant effect would also contribute to the effects of the heparins. Nevertheless, the presence of anticoagulant property-independent pathway might play an important role, and this speculation was supported by the animal study. Indeed, argatroban did not show the organ protective effects under the same setting. As for the anticoagulant effect, Xu et al. [2] were concerned about the increase of bleeding incidence by activated protein C. Similarly, we were afraid of this problem with heparins.

In vitro experiment was performed in a coagulation factor-free medium, and the effect of argatroban could not be expected. Tsen et al. [26] performed the in vitro experiment by using plasma and reported that both UFH and argatroban significantly decreased neutrophil adhesion and platelet-neutrophil aggregation. They concluded that UFH and argatroban decrease sepsis-induced neutrophil-endothelial cell interactions by inhibiting thrombin activity. In our animal study, while the WBC and platelet counts were better maintained in the argatroban group, no organ-protective effect was observed in this group. The reason is not clear; however, we suppose that the presence or absence of anti-histone property may concern to this difference. Tanaka et al. [27] also reported that while the protective effect was seen following treatment with UFH and LMWH, it was not seen in the sepsis models treated with argatroban.

Besides activating the coagulation cascade, histones also activate platelets [28]. Siedel et al. [29] reported the protective effects of antiplatelet therapy in an animal model of sepsis. In regard to antiplatelet therapy, we also examined the effect of ticlopidine in the same model that was used in the present study; however, no beneficial effect was recognized (data not shown).

Finally, there were some limitations of this study. First, although we selected the doses of UFH and LMWH that exert comparable anticoagulant activity, the doses were much higher than the clinical settings and thus, the results cannot be directly reflected to the clinics. We have to realize that the incidence of bleeding must increase with these high-dose heparins. Therefore, the effects of heparins should be examined in lower doses in the next step. Second, although the survival rate was improved by heparins, we could not reach the definitive conclusion since the number of animals was limited. Third, we used only histone H3 in this experiment; however, histone H4 rather than H3 should be examined in the future study. Fourth, we used coagulation factor-free medium in the in vitro study to examine the direct cytotoxicity of histone H3, because it is known that the toxic effects of histone are significantly attenuated by plasma proteins [30]. Several proteins related to the coagulation cascade, such as fibrinogen and fibrinogen degradation products, have been reported to interact with histones [31]. Therefore, the effect of the anticoagulants of protecting against cell death should also be examined in the presence of plasma.

## Conclusions

Both UFH and LMWH attenuated the toxicity of histone H3, in vivo as well as in vitro. The effects of heparins shown in ex vivo study were independent of their anticoagulant effect. The effects of UFH and LMWH were comparable in the present study. In contrast to the heparins, although argatroban suppressed the decrease of the WBC and platelet counts, it failed to exhibit any organ-protective effects.
